# Predictors influencing neurodevelopment during the infancy of term infants

**DOI:** 10.3389/fped.2025.1581682

**Published:** 2025-10-28

**Authors:** Yixia Zhang, Bo Hu, Zhipeng Jin, Shaowen Wang, Qingqing Wang, Jiyu Nie

**Affiliations:** ^1^ Child Health Care, Children's Hospital Affiliated to Zhengzhou University, Zhengzhou, Henan, China; ^2^ Pediatric Intensive Care Unit, Children's Hospital Affiliated to Zhengzhou University, Zhengzhou, Henan, China

**Keywords:** neurodevelopment, term infants, infancy, gestational age, birth weight

## Abstract

**Objectives:**

This study aimed to determine the influencing factors of neurodevelopment of term infants (37–41 6/7 weeks) aged <1 year to provide a basis for neurodevelopment monitoring and management of term infants.

**Study design:**

A total of 327 term infants aged 4–12 months who visited the outpatient department of Child Health Care at the Children’s Hospital Affiliated to Zhengzhou University from December 2023 to June 2024 were included. The Developmental Behavior Assessment Scale for Children aged 0–6 years was used to assess the neurodevelopment of infants who underwent routine physical examination. Univariate and multiple linear regression analyses of the influencing factors on neurodevelopment were conducted.

**Results:**

Greater gestational age (GA) and birth weight (BW) were independent predictors of a higher total development quotient (TDQ) score (*t* = 2.191 and 2.462, respectively; both *p* < 0.05). Overweight and a trend toward overweight in infants were predictors of a low TDQ score (*t* = −2.663, *p* = 0.008). The *R*^2^ value was 0.135 (adjusted *R*^2^ = 0.116) with a root mean square error (RMSE) of 7.138, showing that 13.5% of the TDQ score is explained by differences in GA, BW, and body mass index (BMI). Increased GA and maternal folic acid supplementation prior to pregnancy were independently associated with a higher gross motor score (*t* = 2.377 and −2.128, respectively; both *p* < 0.05). Conversely, infant overweight status or a trend toward overweight was associated with a lower gross motor score (*t* = −2.466, *p* = 0.014). Greater GA and older age were independent predictors of a higher fine motor score (*t* = 2.155 and 4.502, respectively; both *p* < 0.05). Longer days of neonatal intensive care unit (NICU) admission during the neonatal period were a predictor of a lower fine motor score (*t* = −3.528, *p* < 0.001). Greater GA was an independent predictor of a higher adaptability score (*t* = 3.245, *p* < 0.001). Older age was a predictor of a lower adaptability score (*t* = −4.113, *p* < 0.001). Maternal junior college or above was an independent predictor of a higher language score (*t* = 2.350, *p* = 0.019). Older age and gestational hypertension were predictors of a lower language score (*t* = −5.553 and −2.604, respectively; both *p* < 0.05). No factor was found to be an independent predictor of the social behavior score.

**Conclusion:**

GA, BW, BMI, days of NICU admission, prenatal folic acid supplementation of mothers, gestational hypertension, and maternal educational level were influencing factors in the neurodevelopment of infants aged 4–12 months.

## 1 Introduction

Infants are susceptible to neonatal diseases and adverse pregnancy factors, both of which can affect their neurodevelopment (1). The level of neurodevelopment in infants is crucial to their life course and growth trajectory (2), with long-term effects on health, well-being, and earning potential in adulthood (3). More than 40% of children's neurodevelopment has not reached its potential level of neurodevelopment (4). Rapid differentiation in the visual, auditory, and motor cortices, limbic system, and small brain cells during infancy is essential for building neural structural connections that enable more complex behaviors and functions later in life (5, 6). The brain development of infants occurs during a highly sensitive period characterized by strong plasticity. Early identification of children at risk of neurodevelopmental delay, along with systematic stimulation in early infancy, may be beneficial for building neuronal networks and improving cognitive outcomes during this critical period (7). Consequently, it can effectively improve the level of children's neurodevelopment and maximize the potential of individual abilities of children to the fullest extent (8, 9).

Most previous studies primarily focused on the factors influencing the neurodevelopmental outcomes of premature infants (10–13). Additionally, studies on the neurodevelopment of term infants have mostly compared their outcomes with those of premature infants (14, 15). Due to the relatively low incidence of various diseases in term infants, the risk factors affecting neurodevelopment in these infants are often overlooked. The data on factors influencing neurodevelopment outcomes in term infants are very limited, making it crucial to identify adverse factors related to infants' neurodevelopment at an early stage. In the present study, we examined the influencing factors of neurodevelopment, focusing on the infancy of term infants. This study provides a basis for management and early intervention for term infants.

## 2 Subjects and methods

### 2.1 Subjects

This study was a cross-sectional survey. A total of 327 term infants aged 4–12 months who visited the outpatient department of Child Health Care at the Children's Hospital Affiliated to Zhengzhou University from December 2023 to June 2024 were included. In our outpatient clinic, infants under 1 year old can undergo developmental screening using either the Developmental Behavior Assessment Scale for Children aged 0–6 years (Child Assessment Scale-II) or the Ages and Stages Questionnaires (ASQ). The ASQ is a parent-completed questionnaire that is concise yet somewhat subjective, covering five developmental domains, namely, communication, gross motor, fine motor, problem-solving, and personal–social skills. The developmental quotient (DQ) assessment is administered by trained professional evaluators. Based on our clinical experience, infants above 4 months of age demonstrate better interactive capacity; therefore, we prioritized DQ assessments for this age group.

Inclusion criteria: no physical disabilities, no abnormalities in visual and auditory tracking, and informed consent from family members. Exclusion criteria: infants with missing data, infants with limited activity due to trauma or surgery within the past month, and infants diagnosed with genetic metabolic diseases or central nervous system diseases, such as intracranial tumors and encephalitis.

### 2.2 Basic information collected by questionnaire survey

Basic information of infants included consultation card number, age, gender, birth weight (BW), gestational age (GA), body mass index (BMI), and whether hospitalization was required for neonatal diseases and birth complications. Basic information about parents included whether the parents supplemented folic acid, health issues of the mother during pregnancy, smoking and drinking habits of parents, and the educational level of parents and caregivers. Questionnaires were administered by a trained outpatient physician or completed by parents under the guidance of a trained physician assistant. The purpose and significance of the survey were explained to parents.

In our cohort, neonatal diseases and maternal pregnancy complications were defined as follows:

Hypoglycemia (16): For neonates with high-risk factors for hypoglycemia, routine bedside glucose monitoring using a glucometer is performed. For neonates without high-risk factors, glucose monitoring is not routinely conducted; however, if signs or symptoms suggestive of neonatal hypoglycemia appear, immediate glucose testing is required. A blood glucose level below 2.2 mmol/L is diagnostic of neonatal hypoglycemia.

Pathological jaundice (16): (1) Jaundice appearing within 24 h after birth, with total serum bilirubin (TSB) >102 μmol/L (6 mg/dL). (2) Term infants, TSB >220.6 μmol/L (12.9 mg/dL); preterm infants, TSB >255 μmol/L (15 mg/dL). (3) Direct (conjugated) bilirubin >26 μmol/L (1.5 mg/dL). (4) TSB rising >85 μmol/L (5 mg/dL) per day. (5) Prolonged jaundice lasting beyond 2–4 weeks or progressive worsening.

Neonatal Pneumonia (16): The diagnosis requires either pulmonary consolidation on physical examination or persistent medium/fine moist rales on lung auscultation, combined with radiographic evidence of inflammatory infiltrates on chest imaging, along with the presence of supporting respiratory symptoms such as cough, fever, or tachypnea.

Gestational hypertension (17): Blood pressure criteria for hypertension in pregnancy were based on American Heart Association (AHA)/American College of Cardiology (ACC) definitions. It is defined as a systolic blood pressure (SBP) of 140 mmHg or more, a diastolic blood pressure (DBP) of 90 mmHg or more, or both after 20 weeks of gestation.

Gestational diabetes mellitus (GDM) (18): During weeks 24–28 of pregnancy, a fasting plasma glucose level ≥5.1 mmol/L can directly establish the diagnosis of GDM. For the 75 g oral glucose tolerance test, GDM is diagnosed if any one of the following values is met or exceeded: (1) fasting, ≥5.1 mmol/L; (2) 1 h postprandial, ≥10.0 mmol/L; (3) 2 h postprandial, ≥8.5 mmol/L.

Hypothyroidism during pregnancy: According to the diagnostic criteria of hypothyroidism during pregnancy (19), patients with hypothyroidism were divided into the following: the (1) overt hypothyroidism group, serum thyroid-stimulating hormone (TSH) >3.6 mIU/L and FT4 decreased, or serum TSH >10 mIU/L regardless of whether FT4 was normal or not; the (2) subclinical hypothyroidism group, serum TSH >3.6 mIU/L, the serum FT4 level was normal; the (3) low T4 group, the TSH level was normal, but the serum FT4 level was lower than normal.

Anemia during pregnancy (20): Maternal anemia is defined as hemoglobin <11 g/dL in the first/third trimester or <10.5 g/dL in the second trimester per WHO standards.

### 2.3 Neurodevelopment assessment

The neurodevelopment of the infants was evaluated using the Child Assessment Scale-II with a standardized toolbox. The assessment can be completed within approximately 30 min (the detailed methodology is provided in Supplementary File 1). The Child Assessment Scale-II is performed by a trained and qualified pediatrician and includes 8–10 evaluation items for each monthly age cohort (the developmental scale for children aged 0–6 years is provided in Supplementary File 2). This scale is a healthcare industry standard issued by the National Health and Family Planning Commission of the People's Republic of China. It has been widely adopted as a standardized diagnostic instrument throughout China. This scale demonstrates excellent reliability and validity across all age groups, with Cronbach's *α* coefficients ranging from 0.86 to 0.91 for the full scale. It also shows strong discriminative validity and high test–retest (21, 22).

The DQ is the main index (DQ = mental age/chronological age × 100) used to measure children's total neurodevelopment (TDQ) and specific domains of neurodevelopment, including gross motor, fine movement, adaptability, language, and social behavior (23). All assessments were conducted in a standardized measuring room, where the environment and facilities meet the assessment requirements. A lower DQ score represents a lower level of neurodevelopment.

### 2.4 Statistical analysis

The data were analyzed using the IBM SPSS/WIN 27.0 program (IBM Corporation, Armonk, NY, USA). The Shapiro–Wilk normality test was performed. The data for the normal distribution were expressed as mean ± standard deviation, whereas the data for the non-normal distribution were described as median and interquartile range. Pearson’s correlation analysis was used when the two continuous variables fit a normal distribution, and Spearman’s correlation analysis was used when they did not fit a normal distribution. Comparison of non-normally distributed measures between the two groups was performed using the Mann–Whitney *U* non-parametric test, while the Kruskal–Wallis *H* non-parametric test was used for comparisons among multiple groups. Comparison of normally distributed measures between the two groups was performed using the independent samples *t*-test, and one-way analysis of variance (ANOVA) was used for comparisons among multiple groups. Linear regression was used to analyze the influencing factors of neurodevelopment during the infancy of term infants. Multiple linear regression analysis was performed on the indicators that showed statistical significance in the single-factor analysis. No multicollinearity was observed in the multiple regression analysis. *P*-values <0.05 were considered statistically significant.

### 2.5 Ethical considerations

This study was approved by the Children's Hospital Affiliated to Zhengzhou University Ethics Review Committee (No. 2022-K-92). The purpose, contents, and research procedures of the study were described and explained to all eligible parents of participants. The participants were assured that their data would be processed anonymously, that no personal or identifiable information would be exposed, and that the data would be used solely for research purposes.

## 3 Results

### 3.1 General description of the term infants

**Figure 1:**
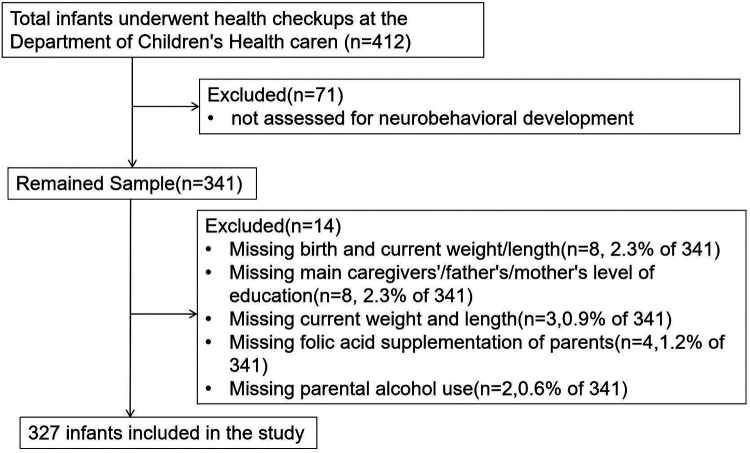
Flow diagram of enrolled infants.

A total of 412 infants who fulfilled the eligibility criteria were screened, and a final sample of 327 were enrolled (Figure 1). Of these, 188 were male (57.5%) and 139 were female (42.5%), all of whom underwent health checkups at the Department of Child Health Care, Children's Hospital Affiliated to Zhengzhou University. The infants had an average GA of 39.3 (38.6, 40.0) weeks and an average BW of 3.3 (3.0, 3.5) kg. A total of 43 infants (13.1%) were classified as overweight. Caesarean delivery was the predominant delivery method, accounting for 56.9%, while vaginal delivery accounted for 43.1%. There were 184 infants (56.3%) whose primary caregivers were their parents.

### 3.2 The self-influencing factors of neurodevelopment in infancy

There were significant differences in the TDQ in terms of the BW, GA, BMI, and days of neonatal intensive care unit (NICU) admission during the neonatal period and whether accompanied by hypoglycemia or jaundice during the neonatal period (*r* = 0.129, *r* = 0.223, *H* = 0.610, *F* = 6.985, *t* = −2.220, and *t* = −3.221; all *p* < 0.05). Older GA was associated with higher TDQ. Larger GA and BW, an appropriate BMI for the infants, shorter NICU admission during the neonatal period (<7 days or no hospitalization), and the absence of hypoglycemia or jaundice during the neonatal period were associated with a higher TDQ score. There were no significant differences in TDQ scores in terms of gender, delivery model, length at delivery, length for age, weight for age, time spent in outdoor activity/free play/infants were held, and whether infants were accompanied by pneumonia during the neonatal period (all *p* > 0.05) (Tables 1, 2).

**Table 1:** The self-influencing factors of neurobehavioral development in term infants (between-group analysis).

Factors	TDQ score	Test statistic	*P*
Gender (frequency)		−1.535^a^	0.125
Male (188)	88.7 ± 7.6		
Female (139)	90.3 (84.8, 94.7)		
Type of delivery (frequency)		−0.143^b^	0.887
Vaginal delivery (141)	88.9 ± 8.0		
Cesarean section (186)	89.1 ± 7.3		
LAZ (frequency)		0.603^c^	0.548
Length within normal range (274)	89.1 ± 7.2		
Growth retardation (16)	87.0 ± 10.2		
Tall stature (34)	89.4 ± 9.0		
WAZ (frequency)		0.382^c^	0.683
Weight within normal range (250)	89.2 ± 7.6		
Low infant weight (45)	88.4 ± 8.4		
Overweight or obese (32)	88.2 ± 6.1		
BMI (frequency)		10.610^d^	**0**.**005**
Weight within normal range (198)	90.9 (86.5, 94.9)		
Trends in overweight (88)	87.8 ± 7.8		
overweight (43)	87.0 ± 6.2		
Days of NICU admission (days)		6.985^c^	**0**.**001**
0 (266)	89.7 ± 7.1		
<7 days (32)	87.0 ± 8.1		
≥7 days (29)	84.8 ± 9.8		
Neonatal hypoglycemia (frequency)		−2.22^b^	**0**.**027**
Yes (8)	83.2 ± 10.4		
No (319)	89.2 ± 7.5		
Neonatal jaundice (frequency)		−3.221^b^	**0**.**001**
Yes (60)	86.2 ± 8.0		
No (267)	89.6 ± 7.4		
Neonatal pneumonia (frequency)		−1.538^b^	0.125
Yes (13)	85.8 ± 8.9		
No (314)	89.1 ± 7.5		

TDQ, total development quotient score; BW, birth weight; GA, gestational age; BMI, body mass index (13.7–17.8 kg/m^2^, weight within normal range; >17.8–19.4 kg/m^2^, trend toward overweight; >19.4 kg/m^2^, overweight) (24, 25); LAZ, *Z*-score of length-for-age (−2 to 2, length within normal range; less than −3, growth retardation; >2, tall stature); WAZ, *Z*-score of weight-for-age (−1 to 2, weight within normal range; less than −1, low infant weigh; >2, overweight or obese) (25).

The bold values indicate that the differences are statistically significant.

^a^ Represent Mann–Whitney *U* non-parametric tests.

^b^ Represent independent samples *t*-test.

^c^ Represent one-way ANOVA.

^d^ Represent Kruskal–Wallis *H* non-parametric test.

**Table 2:** The self-influencing factors of neurobehavioral development in term infants (correlation analysis).

Factors	Median (upper quartile, lower quartile)	TDQ score	Test statistic	*P*
Age (month)	8.0 (6.0, 10.0)	89.0 ± 7.6	−0.229^a^	0.819
BW (kg)	3.30 (3.00, 3.50)	89.0 ± 7.6	0.129^a^	**0**.**019**
Length at delivery (cm)	50.0 (49.0, 51.0)	89.0 ± 7.6	0.059^a^	0.285
GA (week)	39.3 (38.6, 40.0)	89.0 ± 7.6	0.223^a^	**<0**.**001**
Outdoor activity (h)	1.5 (1.0,2.5)	89.0 ± 7.6	0.015^a^	0.791
Free play (h)	3.0 (2.5,5.0)	89.0 ± 7.6	0.038^a^	0.494
Infants are held (h)	4.0 (3.0,5.5)	89.0 ± 7.6	−0.007^a^	0.899

TDQ, total development quotient score; BW, birth weight; GA, gestational age.

The bold values indicate that the differences are statistically significant.

^a^ Represents Pearson’s correlation analysis.

### 3.3 The parental or caregiver influencing factors of neurodevelopment in term infants

There were significant differences in the TDQ in terms of maternal educational level (*F* = 4.483, *p* = 0.012), with higher maternal educational level associated with higher TDQ scores. No significant differences in the TDQ were observed in terms of maternal adversity during pregnancy (gestational hypertension, GDM, hypothyroidism, anemia), parental smoking and alcohol use, folic acid supplementation of parents, and educational level of fathers or caregivers (all *p* > 0.05) (Table 3).

**Table 3:** The parental or caregiver influencing factors of neurobehavioral development in term infants (between-group analysis).

Factors	Frequency (*n*)	TDQ score	Test statistic	*P*
Gestational hypertension (frequency)			−1.707^a^	0.089
	Yes (10)	85.0 ± 9.8		
	No (317)	89.1 ± 7.5		
Gestational diabetes mellitus (frequency)			−0.743^a^	0.458
	Yes (32)	88.1 ± 6.7		
	No (295)	89.1 ± 7.7		
Hypothyroidism during pregnancy (frequency)			−0.865^a^	0.388
	Yes (39)	89.0 ± 7.1		
	No (288)	88.9 ± 7.7		
Anemia during pregnancy (frequency)				
	Yes (94)	89.1 ± 6.7	0.057^a^	0.955
	No (233)	89.0 ± 7.9		
Folic acid supplementation of fathers (frequency)			1.480^b^	0.229
	Regular (33)	91.1 ± 9.6		
	Intermittent (22)	88.0 ± 5.3		
	No (273)	88.8 ± 7.5		
Prenatal folic acid supplementation of mothers (frequency)			3.627^a^	0.163
	Regular (202)	89.7 ± 7.4		
	Intermittent (55)	88.9 ± 7.3		
	No (70)	88.6 (81.6, 93.3)		
Folic acid supplementation during pregnancy (frequency)			2.272^b^	0.105
	Regular (161)	89.8 ± 6.8		
	Intermittent (50)	89.2 ± 8.1		
	No (116)	87.8 ± 8.3		
Smoking of mothers (frequency)			−4.61^a^	0.645
	Yes (8)	87.8 ± 8.4		
	No (319)	89.0 ± 7.6		
Smoking of fathers (frequency)			−0.88^c^	0.374
	Yes (127)	89.2 (84.2, 93.7)		
	No (200)	89.6 ± 7.6		
Drinking of mothers (frequency)			1.516^a^	0.130
	Yes (25)	91.2 ± 6.6		
	No (302)	88.8 ± 7.7		
Drinking of fathers (frequency)			0.685^ta^	0.494
	Yes (132)	89.4 ± 7.1		
	No (195)	88.8 ± 7.9		
Father's educational level (frequency)			2.746^b^	0.066
High school and below (46)	86.6 ± 7.3			
Junior college (83)	89.6 ± 8.4			
Bachelor’s degree or above (198)	89.5 ± 7.2			
Maternal educational level (frequency)				
High school or below (40)	85.7 ± 9.7	4.483^F^	**0**.**012**	
Junior college (91)	89.1 ± 6.9			
Bachelor’s degree or above (196)	89.7 ± 7.3			
Educational level of caregiver (frequency)				
High school or below (163)	88.5 ± 7.7	0.759^F^	0.469	
Junior college (63)	89.8 ± 7.4			
Bachelor’s degree or above (101)	89.3 ± 7.6			
Primary caregiver (frequency)			0.044^b^	0.957
	Parents (184)	89.1 ± 8.0		
	Grandparents (128)	89.0 ± 7.1		
	Others (15)	88.5 ± 7.0		

TDQ, total development quotient score. Mean ± standard deviation: normal distribution. Median and interquartile range: non-normal distribution.

The bold values indicate that the differences are statistically significant.

^a^ Represents independent-sample *t*-test.

^b^ Represents one-way ANOVA.

^c^ Represents Mann–Whitney *U* non-parametric tests.

### 3.4 Multivariate linear regression analysis of influencing factors of neurodevelopment in term infants

Multivariate linear regression analysis using the forward stepwise method was conducted with the TDQ score as the dependent variable. The independent variables included factors found to be significant in the univariate analysis, such as GA, BW, BMI of infants, days of NICU admission during the neonatal period, whether the infants were accompanied by hypoglycemia or jaundice during the neonatal period, and the maternal educational level. It was found that greater GA and BW were independent predictors of a higher TDQ score (*t* = 2.191 and 2.462, respectively; both *p* < 0.05). Overweight or a trend toward overweight of infants was a predictor of low TDQ score (*t* = −2.663, *p* = 0.008) (Table 4). The multifactor linear regression model was statistically significant (*F* = 7.102, *p* < 0.001). The *R*^2^ value was 0.135, showing that 13.5% of the TDQ score is explained by differences in GA, BW, and BMI.

**Table 4:** Multivariate analysis of TDQ score of term infants.

Model	Coefficients B	Beta	*t*	*P*
Constant	45.144		3.142	0.002
GA	0.863	0.130	2.191	0.029
BW	2.699	0.143	2.462	0.014
BMI	−1.516	−0.143	−2.663	0.008

GA, gestational age; BW, birth weight; BMI, body mass index (13.7–17.8 kg/m^2^, weight within normal range; >17.8–19.4 kg/m^2^, trend toward overweight; >19.4 kg/m^2^, overweight) (24, 25).

The same statistical methods were carried out as TDQ. Multivariate linear regression analysis by forward stepwise method was carried out using gross motor, fine motor, adaptability, language, and social scores as dependent variables and factors found significant on univariate analysis as independent variables. It was found that greater GA and maternal folic acid supplementation before pregnancy were independent predictors of a higher gross motor score (*t* = 2.377 and −2.128, respectively; both *p* < 0.05). Overweight or a trend toward overweight of infants was a predictor of a low gross motor score (*t* = −2.466, *p* = 0.014) (Table 5). The *R*^2^ value was 0.158, showing that 13.5% of the TDQ score is explained by differences in GA, BW, and BMI. Greater GA and older age were independent predictors of a higher fine motor score (*t* = 2.155 and 4.502, respectively; both *p* < 0.05). Longer days of NICU admission during the neonatal period was a predictor of a lower fine motor score (*t* = −3.528, *p* < 0.001) (Table 5). Greater GA and young age were independent predictors of a higher adaptability score (*t* = 3.245 and −4.113, respectively; both *p* < 0.001) (Table 5). The multifactor linear regression model was statistically significant (*F* = 7.102, *p* < 0.001). The *R*^2^ value was 0.135, showing that 13.5% of the TDQ score is explained by differences in GA, BW, and BMI. Greater GA and older age were independent predictors of a higher fine motor score (*t* = 2.155 and 4.502, respectively; both *p* < 0.05). Longer days of NICU admission during the neonatal period were an independent predictor of a lower fine motor score (*t* = −3.528, *p* < 0.001) (Table 5). The multifactor linear regression model was statistically significant (*F* = 7.102, *p* < 0.001). The *R*^2^ value was 0.135, showing that 13.5% of the TDQ score is explained by differences in GA, BW, and BMI. Maternal junior college or above was an independent predictor of a higher language score (*t* = 2.350, *p* = 0.019). Older age and gestational hypertension were independent predictors of a lower language score (*t* = −5.553 and −2.604, respectively, both *p* < 0.05) (Table 5). The multifactor linear regression model was statistically significant (*F* = 7.102, *p* < 0.001). The *R*^2^ value was 0.135, showing that 13.5% of the TDQ score is explained by differences in GA, BW, and BMI.No factor was found to be an independent predictor of social behavior score.

**Table 5:** Multivariate analysis of the gross motor, fine movement, adaptability, and language score of term infants.

Model	Coefficients *B*	Beta	*t*	*P*
Gross motor score				
Constant	45.651		1.454	0.147
Prenatal folic acid supplementation of mothers	−2.091	−0.138	−2.128	0.034
BW	5.299	0.172	2.377	0.018
BMI	−2.343	−0.136	−2.466	0.014
Fine movement score				
Constant	25.426		1.182	0.238
GA	1.266	0.124	2.155	0.032
Age	1.108	0.234	4.502	<0.001
Days of NICU admission	−3.452	−0.181	−3.528	<0.001
Adaptability score				
Constant	35.595		1.748	0.081
Age	−0.983	−0.220	−4.113	<0.001
GA	1.684	0.175	3.245	<0.001
Language score				
Constant	92.803		25.397	<0.001
Age	−1.437	−0.294	−5.553	<0.001
Gestational hypertension	−9.623	−0.137	−2.604	0.010
Maternal educational level	2.124	0.123	2.350	0.019

GA, gestational age; BW, birth weight; BMI, body mass index (13.7–17.8 kg/m^2^, weight within normal range; >17.8–19.4 kg/m^2^, trend toward overweight; >19.4 kg/m^2^, overweight) (17, 18).

### 3.5 *Post hoc* power analysis

*Post hoc* power analysis was calculated using G*Power 3.1 (linear multiple regression, *α* = 0.05, 31 predictors) based on the observed effect size (*f*^2^ = 0.156 derived from *R*^2^ = 0.135 [*f*^2^ = *R*^2^/(1 − *R*^2^) = 0.135/0.865 = 0.156]). The analysis demonstrates that our current sample size of 327 achieves 98.8% statistical power (exceeding the conventional 80% threshold). Although the model's explanatory power (*R*^2^ = 0.135) reflects the inherent complexity of predictors influencing neurodevelopment during the infancy of term infants, all key predictors showed statistical significance (*P* < 0.05) with clinically meaningful effect sizes.

## 4 Discussion

Our study has evaluated the heterogeneity of neurodevelopment during infancy among individuals born beyond 37 gestational weeks. This cross-sectional study found that the TDQ, fine motor, and adaptability scores of term infants increased with GA, which was consistent with the research of Wang et al. (26), Murray et al. (27), and Hua et al. (28). This challenges our perception of term infants. Previously, it was thought that term children born at 37–41 6/7 weeks of GA would have similar developmental trajectories and health outcomes. However, the brain is only 90% of full-term (GA ≥ 39 weeks) weight even at 38 weeks of gestation (29). The development of neural connections in certain areas of the brain of term infants improves with increasing GA, which may contribute to the effect of GA on neurodevelopment during infancy (30).

GA is a key indicator for assessing fetal growth, determined by the interaction of genetics and the intrauterine environment, and is associated with multiple health outcomes later in life (31). Kirkegaard et al. (32) found that higher BW was associated with a higher intelligence quotient at 5 years of age. Our research found that the level of neurodevelopment in infancy is also affected by BW, specifically, the total developmental quotient (TDQ) and gross motor score increase with BW, which is consistent with the research of Zhang et al. (33).

There is a positive association between BMI in infants and BMI in children and adolescents (34). It is well established that childhood obesity or overweight is associated with cognitive impairment (35). Our study found that being overweight had adverse effects on infants' TDQ and gross motor, which was consistent with the research of Xiong et al. (36). Higher BMI was associated with imaging metrics of poorer brain structure and connectivity as well as hindered interval development (37). Gut dysbiosis in obese children may affect cognitive function by influencing the volume of their cerebral cortex (38, 39). Similar mechanisms may be involved in obese infants, and further studies are needed.

Our study shows that the gross motor scores of infants whose mothers received prenatal folic acid supplementation were significantly lower than those of infants whose mothers did not receive prenatal folic acid supplementation, which was similar to the research of Huang et al. (40). Folic acid supplementation increased the level of folic acid and reduced homocysteine levels in the brain tissue of offspring (41, 42), thereby decreasing the wrong incorporation of uracil into telomeres, and protected *de novo* telomere synthesis of offspring. This was beneficial for the development of early sensory–motor function, spatial learning, and memory in adolescence and adulthood (41).

The results show that infants with longer days of NICU admission have lower fine movement scores during the neonatal period. Infants may be exposed to neurotoxic chemicals, medications, and built environments (excess light and loud noise) that can cause pain and stress when admitted to the NICU (43, 44). Both the built environment and NICU care practices that may cause pain and stress are known to activate the hypothalamic–pituitary–adrenal axis (45). Such stress responses have a negative impact on brain development.

The results show that the language scores of infants with gestational hypertension were significantly lower than those of infants without gestational hypertension, which was consistent with the research of Palatnik et al. (46) and Whitehouse et al. (47). Hypertension during pregnancy causes vasoconstriction, leading to hypoxia in the placental environment. Both acute and chronic hypoxia may disrupt placental signaling, impairing the brain structures of the offspring. These mechanisms can have potentially negative impacts on neural pathways associated with speech and language abilities (48). A higher maternal educational level was associated with a higher language score. Higher maternal educational level improves neurodevelopmental outcomes through a favorable socioeconomic status (49). Socioeconomic status affects the level of language development by influencing the activation of language processing areas, specifically the inferior frontal gyrus and superior temporal gyrus (50).

## 5 Strengths and limitations

This study provides important insights into the neurodevelopmental heterogeneity among term infants aged 4–12 months, identifying key influencing factors including GA, BW, BMI, duration of NICU admission, maternal folic acid supplementation before pregnancy, gestational hypertension, and maternal education level. The strength of this research lies in its specific focus on the traditionally overlooked low-risk term infant population, providing important evidence for neurodevelopmental monitoring in these “healthy” infants. The use of standardized neurodevelopmental assessment tools and comprehensive statistical analyses (including *post hoc* power analysis confirming 98.8% statistical power) significantly enhanced the reliability of the findings.

However, several limitations should be acknowledged. First, the retrospective design limited the collection of detailed data for certain variables, such as the duration and clinical manifestations of neonatal hypoglycemia, peak bilirubin levels, and specific treatment modalities for hyperbilirubinemia. The use of dichotomous or trichotomous classifications for parental smoking, alcohol consumption, and folic acid supplementation may have constrained the depth of analysis, as more granular data (e.g., frequency and dosage) could have provided additional insights. Although the current age was adjusted for in the analyses, the relatively wide age range (4–12 months) may obscure age-specific neurodevelopmental patterns. We have provided stratified comparisons (4–6 months/7–9 months/9–12 months) in Supplementary File 3. These results demonstrate that “GA, BW, BMI, days of NICU admission, neonatal pneumonia, and maternal educational level” collectively influenced neurodevelopment across the 4–12 month period. Notably, the specific factors impacting neurodevelopmental outcomes varied across different age subgroups.

No cases of severe neonatal complications (including sepsis, shock, or asphyxia) were documented among the 327 term infants in our cohort. While this finding aligns with the known low incidence of such conditions in this population, it consequently precluded evaluation of their potential impact on neurodevelopmental outcomes. The model's explanatory power was relatively limited (*R*^2^ = 0.135), suggesting that besides the identified factors, other important influences such as sepsis, shock, or asphyxia might not have been captured. Furthermore, the inclusion of infants from only a single-center outpatient setting may limit the generalizability of the findings.

Future research should employ prospective designs combined with standardized data collection protocols to address current limitations. Longitudinal assessments would better capture the dynamic changes in growth indicators and neurodevelopment, while larger sample sizes would facilitate detection of conditions like sepsis, shock, or asphyxia and their effects. More narrowly stratified age group analyses and more detailed exposure assessments would help precisely identify critical developmental windows and risk factors. Despite these limitations, this study underscores the importance of optimizing neurodevelopmental outcomes in term infants through modifiable factors such as maternal health and infant BMI management.

## 6 Conclusions

Our results show the heterogeneity of neurodevelopment among infants born beyond 37 gestational weeks. We found GA, BW, BMI, days of NICU admission, prenatal folic acid supplementation of mothers, gestational hypertension, and maternal educational level are influencing factors in neurodevelopment in infants aged 4–12 months. Prenatal folic acid supplementation of mothers, avoiding non-medically indicated deliveries before 39 weeks, and actively managing the BMI of infants during infancy will benefit the neurodevelopment of infants. It is important to monitor the neurodevelopment of infants who have had neonatal hospitalization, whose mothers have a high school education or below, and those whose mothers have experienced gestational hypertension.

## Data Availability

The raw data supporting the conclusions of this article will be made available by the authors, without undue reservation.
