# Supplementary material for: Predictors influencing neurodevelopment during the infancy of term infants
Source: Front Pediatr. 2025 Oct 28;13:1581682. doi: 10.3389/fped.2025.1581682 (PMC12602470; doi:10.3389/fped.2025.1581682)
Supplement: Supplementary file 1 [file Datasheet1.docx]

**Stratified comparisons (4-6 months/7-9 months/9-12 months)**

Table S1 . Multivariate analysis of TDQ score of term infants (4-6 months).

| Model | Coefficients  B | Beta | *t* | *P* |
| --- | --- | --- | --- | --- |
| Constant | 73.516 |  | 9.705 | < 0.001 |
| BW | 5.423 | 0.236 | -2.274 | 0.024 |
| Days NICU admission | -3.943 | -0.294 | -2.907 | 0.005 |

Table S2. Multivariate analysis of TDQ score of term infants (6-9 months)

| Model | Coefficients  B | Beta | *t* | *P* |
| --- | --- | --- | --- | --- |
| Constant | 24.987 |  | 1.365 | 0.174 |
| GA | 1.644 | 0.283 | 3.526 | < 0.001 |
| Neonatal Pneumonia | -5.129 | -0.182 | -2.274 | 0.024 |

Table S3. Multivariate analysis of TDQ score of term infants（9-12 months）

| Model | Coefficients  B | Beta | *t* | *P* |
| --- | --- | --- | --- | --- |
| Constant | 24.541 |  | 1.033 | 0.304 |
| GA | 1.459 | 0.227 | 2.399 | 0.018 |
| Maternal educational level | 2.376 | 0.992 | 2.395 | 0.019 |
| Neonatal Pneumonia | 9.012 | 0.214 | 2.284 | 0.025 |
