# Supplementary material for: Predictors influencing neurodevelopment during the infancy of term infants
Source: Front Pediatr. 2025 Oct 28;13:1581682. doi: 10.3389/fped.2025.1581682 (PMC12602470; doi:10.3389/fped.2025.1581682)
Supplement: Supplementary file 2 [file Datasheet2.docx]

**Neurodevelopment Assessment Procedure:**

All assessments were performed by qualified examiners, with 8-10 evaluation items included for each monthly age cohort.

1. The primary assessment age (the nominal age closest to the actual age; e.g., 4 months for infants aged 4 months 0 days to 4 months 15 days, or 5 months for those aged 4 months 16 days to 4 months 30 days) serves as the starting point. Testing begins with items corresponding to this primary age.
2. For each developmental domain, regardless of whether the child passes or fails items at their primary assessment age (the nominal age closest to their actual chronological age), the evaluation must systematically extend both forward (to older age groups) and backward (to younger age groups) by two monthly intervals, resulting in five evaluation items included for each monthly age cohort at least.

**For forward testing** (assessing younger age groups), the evaluation of a specific developmental domain is concluded when the child successfully passes all assessment items across two consecutive younger monthly age groups. Should the child fail any single item within these two consecutive younger age groups, the testing must continue to progressively younger ages until all domain-specific items are passed in two consecutive younger age groups, thereby confirming developmental mastery at that level.

**For backward testing** (assessing older age groups), the evaluation of a specific developmental domain is terminated when the child fails all assessment items across two consecutive older monthly age groups. However, if the child passes any single item within these two consecutive older age groups, testing must continue to progressively older ages until all domain-specific items are failed in two consecutive older age groups, thereby establishing the ceiling of developmental attainment for that domain.

3.Following the established calculation rules, developmental scores ≥80 points indicate normal developmental functioning.
